# Cultured pericytes from human brain show phenotypic and functional differences associated with differential CD90 expression

**DOI:** 10.1038/srep26587

**Published:** 2016-05-24

**Authors:** Thomas I-H. Park, Vaughan Feisst, Anna E. S. Brooks, Justin Rustenhoven, Hector J. Monzo, Sheryl X. Feng, Edward W. Mee, Peter S. Bergin, Robyn Oldfield, E. Scott Graham, Maurice A. Curtis, Richard L. M. Faull, P. Rod Dunbar, Mike Dragunow

**Affiliations:** 1 Department of Pharmacology and Clinical Pharmacology, The University of Auckland, Auckland, New Zealand; 2 Centre for Brain Research, The University of Auckland, Auckland, New Zealand; 3 School of Biological Sciences and Maurice Wilkins Centre, The University of Auckland, Auckland, New Zealand; 4 Auckland City Hospital, 1023, Auckland, New Zealand; 5 Lab Plus, 1023, Auckland, New Zealand; 6 Department of Anatomy with Radiology, The University of Auckland, Auckland, New Zealand.

## Abstract

The human brain is a highly vascular organ in which the blood-brain barrier (BBB) tightly regulates molecules entering the brain. Pericytes are an integral cell type of the BBB, regulating vascular integrity, neuroinflammation, angiogenesis and wound repair. Despite their importance, identifying pericytes amongst other perivascular cell types and deciphering their specific role in the neurovasculature remains a challenge. Using primary adult human brain cultures and fluorescent-activated cell sorting, we identified two CD73^+^CD45^−^ mesenchymal populations that showed either high or low CD90 expression. CD90 is known to be present on neurons in the brain and peripheral blood vessels. We found in the human brain, that CD90 immunostaining localised to the neurovasculature and often associated with pericytes. *In vitro,* CD90^+^ cells exhibited higher basal proliferation, lower expression of markers αSMA and CD140b, produced less extracellular matrix (ECM) proteins, and exhibited lesser pro-inflammatory responses when compared to the CD90^−^ population. Thus, CD90 distinguishes two interrelated, yet functionally distinct pericyte populations in the adult human brain that may have discrete roles in neurovascular function, immune response and scar formation.

The central nervous system (CNS) is one of the most vascularised organ systems in our body, yet it remains remarkably immune-privileged due to the presence of the blood-brain barrier (BBB). The BBB consists of endothelial cells, astrocytes and several perivascular cells, including pericytes and mesenchymal stromal cells (MSCs)^1^. Pericytes are perivascular mural cells that are found surrounding endothelial cells and secrete extracellular matrix (ECM) proteins that make up the basement membrane^2^. The brain has the highest concentration of pericytes per vascular endothelial cell^1^,^3^ and they are involved in many facets of vascular function including angiogenesis^4^, vascular stabilisation^5^,^6^, vessel maturation^7^, and perhaps vasoconstriction^8^, although this has been recently challenged^9^. Pericytes also play a considerable role in mediating inflammatory signals both in and out of the CNS^10^,^11^,^12^,^13^,^14^ and some consider pericytes to be one of the ‘brain macrophages’ due to their antigen presenting and phagocytic properties^15^,^16^. Furthermore, pericytes have been implicated in scar formation and fibrosis in neurological conditions such as stroke and spinal cord injury^17^,^18^,^19^. Despite these important functions, there is still controversy over the exact identification of brain pericytes^20^, possibly due to them being largely viewed as a single cell type that supports the function of the vasculature, irrespective of their organ^2^,^3^,^16^,^21^,^22^. However, it is becoming apparent that pericytes play more than a structural role and that they can be very organ specific^3^. This raises the question of whether our brain houses distinct pericyte populations, and if so, what their possible functions might be in brain physiology and pathology.

Many markers are used to identify pericytes, but, unfortunately, none appear to be pericyte-specific. One such marker is CD90/Thy-1, a 25–37 kDa cell surface GPI-anchored glycoprotein that is also expressed in various other cell types, including MSCs, fibroblasts, hematopoietic cells, endothelial cells, and neurons^23^,^24^,^25^. Variations in CD90 expression have been associated with numerous functions that appear context and cell/organ dependent, including neurite outgrowth in neurons^23^,^26^,^27^, cell adhesion in fibroblasts and leukocytes^28^,^29^, and wound repair and fibrosis in fibroblasts and MSCs^30^,^31^. CD90 has also been associated with cancer stem cells in various cancers^13^,^32^,^33^,^34^,^35^,^36^ and its expression in perivascular cells of glioma tissue has been positively correlated with the degree of tumor malignancy^32^,^37^. In addition, CD90^+^ cells reportedly have higher proliferation rates and down-regulate their expression of CD90 upon cellular differentiation and scar formation^38^,^39^,^40^,^41^.

Therefore, we set out to identify whether levels of CD90 expression could distinguish pericyte populations in the adult human brain. Utilising biopsy adult human brain tissue culture, we found CD90 to be a distinguishing marker between two populations of perivascular cells. While both populations had gene expression profiles consistent with pericyte origin, they had differences in functions relevant to the perivascular niche: proliferation, ECM formation, and inflammation.

## Results

### Adult human brain contains CD90^+^ and CD90^−^ perivascular cells

**Figure 1:**
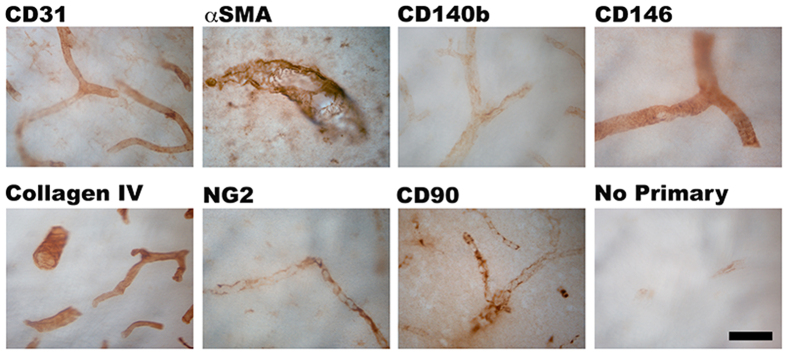
Adult human brain vasculature contains perivascular cells that also express CD90. Photomicrographs of temporal lobe brain sections showing the presence of CD90^+^ staining on neurovasculature amongst other pericyte makers, αSMA, CD140b, CD146 and NG2. Collagen IV staining shows the basement membrane while CD31 staining shows the endothelial cells of the neurovasculature. Scale: 50 μm.

**Figure 2:**
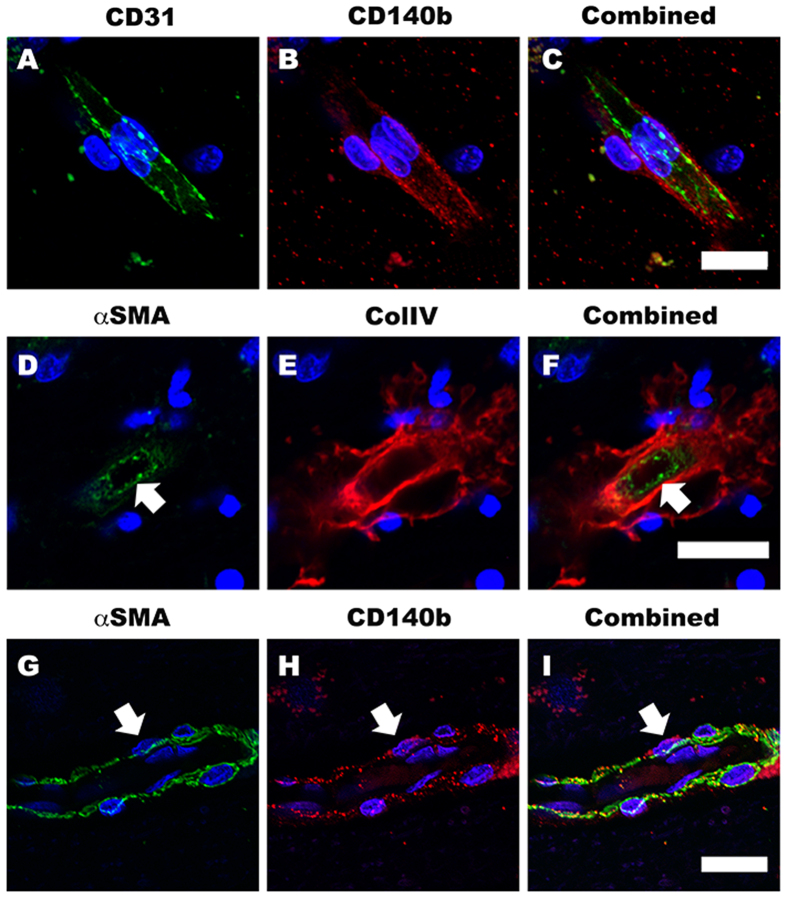
Distinct localisation of BBB cells in the adult human brain vasculature. Confocal triple-labeled immunofluorescent images clearly demonstrate the localization of perivascular cells. CD140b^+^ pericytes were localized on the basal side of the CD31+ endothelial cells (**A–C**). The αSMA expressing pericytes (arrow) were also surrounded by collagen IV positive basement membrane (**D–F**). We also confirmed that αSMA and CD140b were labeling the same pericyte population (**G–I**) arrows). Scale: 10 μm.

**Figure 3:**
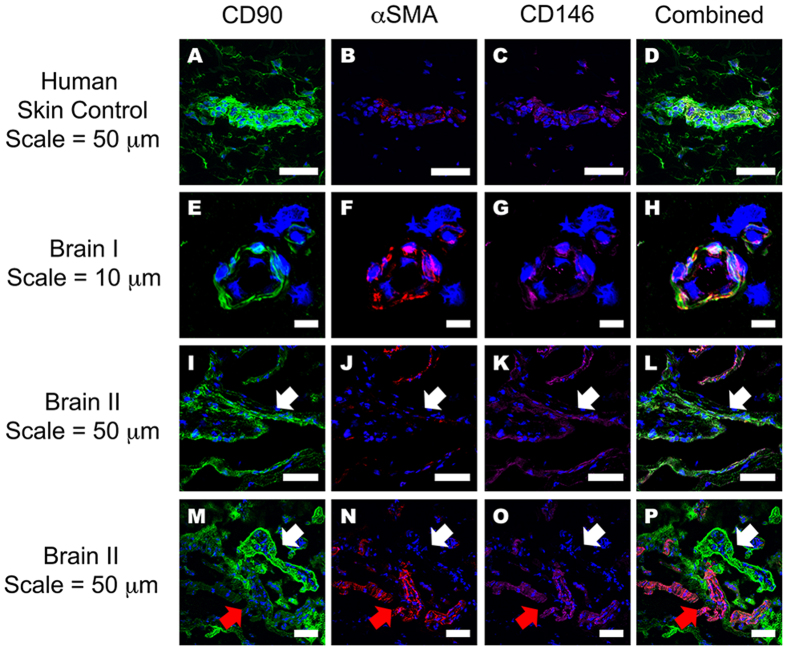
The localisation of both CD90^+^ and CD90^−^ (αSMA^+^CD140b^+^) pericytes around adult human brain vasculature. Confocal quadruple-labeled immunofluorescent images clearly show the localization of the CD90^+^ perivascular cells in the human brain. Adult human dermal skin was used as a positive control, and this shows the presence of CD90^+^ cells surrounding small and large vasculature, as well as in non-vasculature related cells in the parenchyma (**A–D**). Panels (**E–P**) shows representative images from two cases of the five donor brains stained. Many CD90^+^ vessels were also positive for αSMA and CD146 and localised to blood vessels (**E–P**). Furthermore, we found a number of CD90^+^ vessels were not labeled with a pericyte/VSMC marker, αSMA ((**I–P**) white arrows). In some instances, αSMA positive vessels were negative for CD90 ((**M–P**) red arrows). Scale bars ranging from 10 μm to 50 μm are listed on the figure. A total of 5 donor brain specimens and 2 skin specimens were trialed for this experiment.

Despite studies showing CD90^+^ cells in perivascular regions of human glioma^13^,^32^,^37^, there is limited evidence for the presence of CD90^+^ perivascular cells in the non-cancerous adult human brain. To establish their presence in the normal human brain vasculature, we immunohistochemically stained for CD90, along with endothelial (CD31), pericyte (αSMA, CD140b/PDGFRβ, CD146 and NG2) and basement membrane (collagen IV) markers in post-operative human brain tissue. Figure 1 and supplementary Figure 1 show all the above markers localized to their specific regions of the neurovasculature. Colocalisation studies by confocal microscopy revealed that CD31^+^ endothelial cells were surrounded by CD140b^+^ pericytes that were also αSMA^+^ (Fig. 2A–I). These pericytes were further encased by collagen IV positive basement membrane (Fig. 2D–F)^42^,^43^. Figure 3A–D shows that CD90^+^ cells in human skin tissue are strongly localized to regions around the blood vessel, but also on cells in the parenchyma^25^. Unlike in the skin, in the human brain, CD90^+^ cells were exclusively associated with vasculature but did not always co-localize with αSMA^+^ and CD146^+^ cells (Fig. 3E–P). To elucidate the function of CD90^+^ and CD90^−^ cells, they were isolated, sorted and cultured for *in vitro* characterisation and experimentation.

### Primary adult human brain cultures reveal two CD73^+^ populations that can be fluorescent-activated cell sorted into CD90^+^ and CD90^−^ populations and cultured *in vitro*

**Figure 4:**
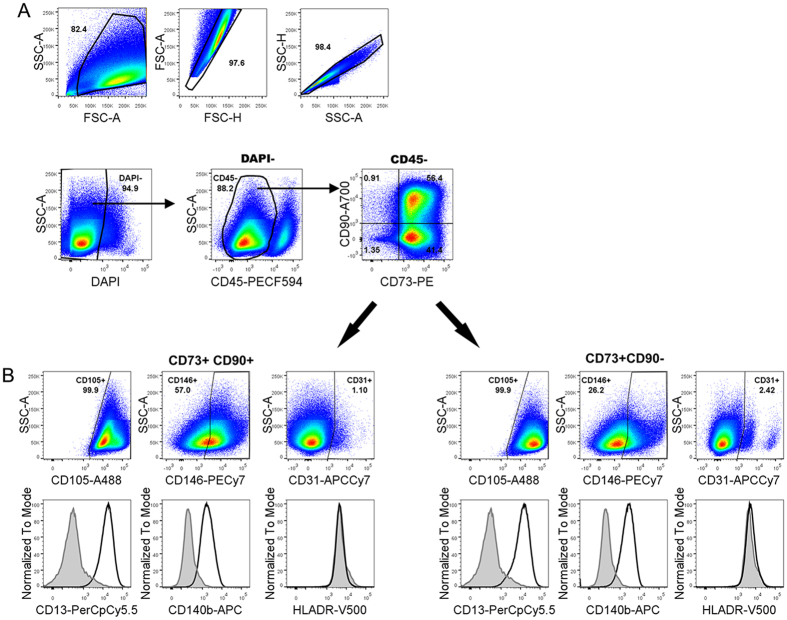
FACS analysis reveals adult human brain perivascular cell cultures consist of two CD73^+^ populations that are CD90^+^ and CD90^−^. Cells isolated from biospy adult human brain tissue were analysed using a 10 colour flow cytometry panel after one passage in culture. The cells were first gated to exclude debris, doublets and nonviable cells (DAPI^+^). CD45^+^ cells were first gated out to assess the non-hematopoietic cell populations. The remaining cells were all CD73^+^, which contained two main populations based on the expression of CD90. Both CD90^+^ and CD90^−^ populations were also positive for CD13, CD146, CD105, and CD140b and negative for HLADR. When comparing the two CD73+ populations, the CD90^+^ cells had lower CD105 and higher CD146 expression, whereas the CD90^−^ population had slightly higher expression of CD140b. The CD90^−^ population also contained a smaller CD146^high^ population compared to the majority of the CD90- population that was low in CD146^+^. Minor CD31^+^ populations were also detectable in both CD73^+^ fractions. Data is representative of 5 donors.

To investigate the phenotypes of the neurovascular cells cultured *in vitro,* adult human brain tissue was cultured for one passage then analyzed and sorted using 10-marker polychromatic flow cytometry. Markers included CD45, CD73, CD90, CD105, CD140b, CD146, CD13, CD31, HLA-DR and DAPI. The cells were gated to exclude debris, doublets and nonviable cells (Fig. 4A). The remaining cells were characterized by first excluding CD45 expression, as this segregated the CD45^+^ microglia/macrophages population from the rest of the culture^44^. The remaining CD45^−^ populations expressed CD73 and further separated into two distinct CD90^+^ and CD90^−^ populations (Fig. 4A). This also confirmed that at least some of the neurovascular cells retained their CD90^+^ phenotype in our culture conditions, although their percentage amongst the CD73^+^ cells varied between cases, ranging from 34–62% (48.0 ± 6.0, n = 5; supplementary Figure 2). Further analysis revealed that the CD45^−^ CD73^+^CD90^+^ population (CD90^+^ hereafter) and the CD45^−^CD73^+^CD90^−^ population (CD90^−^ hereafter) were also CD105^+^ (Fig. 4B). Despite both the CD90^+^ and CD90^−^ populations expressing CD105, the CD90^+^ population generally expressed lower levels of CD105 compared with the CD90^−^ population. In some donors (3 out of 5), there also appeared to be a smaller CD90^+^CD105^high^ population (supplementary Figure 2). Both the CD90^+^ and CD90^−^ populations were positive for CD13 and negative for HLADR. Both populations expressed CD140b; however, expression was always higher in the CD90^−^ population. CD31^low^ and CD31^high^ populations were also present in the CD90^−^ fraction.

**Figure 5:**
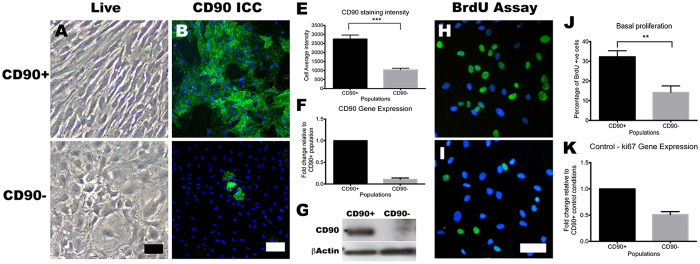
FACS-sorted CD90^+^ and CD90^−^ cells retain their phenotypes *in vitro*. The two FACS sorted CD73^+^ populations exhibited distinct cell morphologies *in vitro*. CD90^+^ cells grew as spindle-shaped cells and in alignment with each other (**A**), while CD90- cells were polygonal in shape and showed no apparent alignment (**C**). CD90 immunolabeling clearly shows the higher expression of CD90 in the CD90^+^ population (**B,D**), and this was quantified using a cell average staining intensity algorithm ((**E**) *p* < 0.001). Gene expression studies using qRT-PCR also corroborates these finding as the CD90^−^ population’s CD90 mRNA expression was~16 fold lower relative to CD90^+^ (**F**). Western blot analysis of CD90 protein also showed a greater level of CD90 in the CD90^+^ population, while the CD90^−^ population had undetectable levels (**G**). Basal cellular proliferation rate was assessed using BrdU assay, which clearly showed a greater BrdU incorporation in the CD90^+^ population compared to the CD90^−^ (**H–J**). Gene expression analysis of the proliferation marker ki67 also indicated a two-fold greater expression in the CD90^+^ population (**K**). Photomicrographs are representative images from 4 different cases while gene expression studies are pooled from at least three cases. Scale: 100 μm. ***p < 0.001 and **p < 0.01.

These two populations of CD73^+^ cells were FACS sorted based on their expression of CD90. The two main populations were cultured to increase cell numbers and further examine their characteristics. In order to investigate whether the CD90 phenotype was maintained in culture post-sort, we re-analysed the cell surface phenotype on some of the cultures after one passage (~10 days). 80 ± 11% (n = 2 cases) of cultured CD90^+^ population retained CD90 expression, while 82 ± 1.0% (n = 2) of the CD90^−^ population remained CD90^−^ for at least one passage (supplementary Figure 3). Figure 5 shows the cellular morphologies of the two populations, and in concordance with previous studies^21^, CD90^+^ cells were smaller in size and spindle-shaped, while CD90^−^ cells were larger and polygonal shaped (Fig. 5A,C). To corroborate our FACS analysis, the CD90^+^ population had much greater CD90 protein expression, as shown by CD90 immunocytochemisty (Fig. 5B,D), staining intensity analysis (Fig. 5E) and western blot analysis (Fig. 5G). Furthermore, CD90 mRNA expression analysis (Fig. 5F) also confirmed the higher expression of CD90 in the CD90^+^ population. The CD90^+^ population also showed greater basal proliferation compared to the CD90^−^ population (Fig. 5H–K). The BrdU assay clearly illustrated higher BrdU incorporation in the CD90^+^ population, as well as higher expression of the cell proliferation marker gene ki67. Taken together, perivascular cell cultures from the adult human brain have two distinct mesenchymal cell types that can be segregated on the basis of CD90 expression and these showed different proliferative potential.

### CD90^+^ and CD90^−^ populations show the greatest differential gene expression in genes regulating cell cycle and ECM production

**Figure 6:**
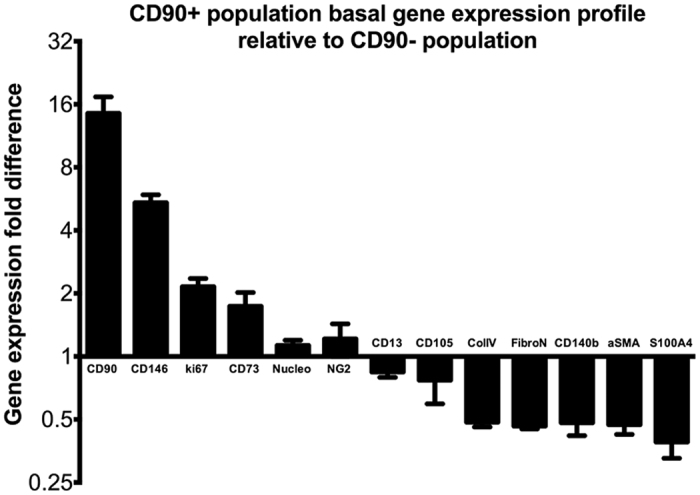
Gene expression profile of mesenchymal, pericyte and extra-cellular matrix protein genes in the CD90^+^ population relative to levels observed in the CD90^−^ population. Graph illustrates the gene expression profile of the CD90^+^ population in reference to the CD90^−^ population. qRT-PCR data from 5 separate experiments from 4 different cases have been compiled in the above data. CD90^+^ population consistently showed greater than two fold higher expression of mesenchymal marker genes CD90, CD146, CD73 and cellular proliferation marker ki67. Conversely, the CD90^−^ population showed greater than two fold higher expression of extracellular matrix and stress-fiber genes such as collagen IV, fibronectin, αSMA and mature pericyte/fibroblast markers CD140b and S100A4.

The two populations were profiled with a panel of mesenchymal cell genes in order to evaluate their gene expression patterns (Fig. 6). Gene expression in each population under basal conditions were measured using qRT-PCR and displayed relative to levels seen in the CD90^−^ population (i.e. no difference in gene expression levels between the two populations would be a value of 1). As expected, the CD90^+^ population had a 16-fold greater level of CD90 gene expression compared to the CD90^−^ population. CD146 and CD73 were 6-fold and 2-fold higher in the CD90^+^ population, respectively. As discussed above, the cell proliferation marker gene ki67 was over two-fold higher in the CD90^+^ population. On the other hand, CD90^−^ population had 2–4 fold higher expression of vascular smooth muscle cell (VSMC) markers when compared to the CD90^+^ population. Specifically, the VSMC marker αSMA, fibroblast-specific protein-1 (S100A4)^45^, and extracellular matrix protein genes collagen IV and fibronectin^46^,^47^, were all 2–3 fold higher in the CD90^−^ population (Fig. 6). CD140b, a classical pericyte marker^17^,^48^, was 2 fold higher in the CD90^−^ cells.

### CD90^−^ cells display a contractile pericyte phenotype that produces greater amounts of extracellular matrix

**Figure 7:**
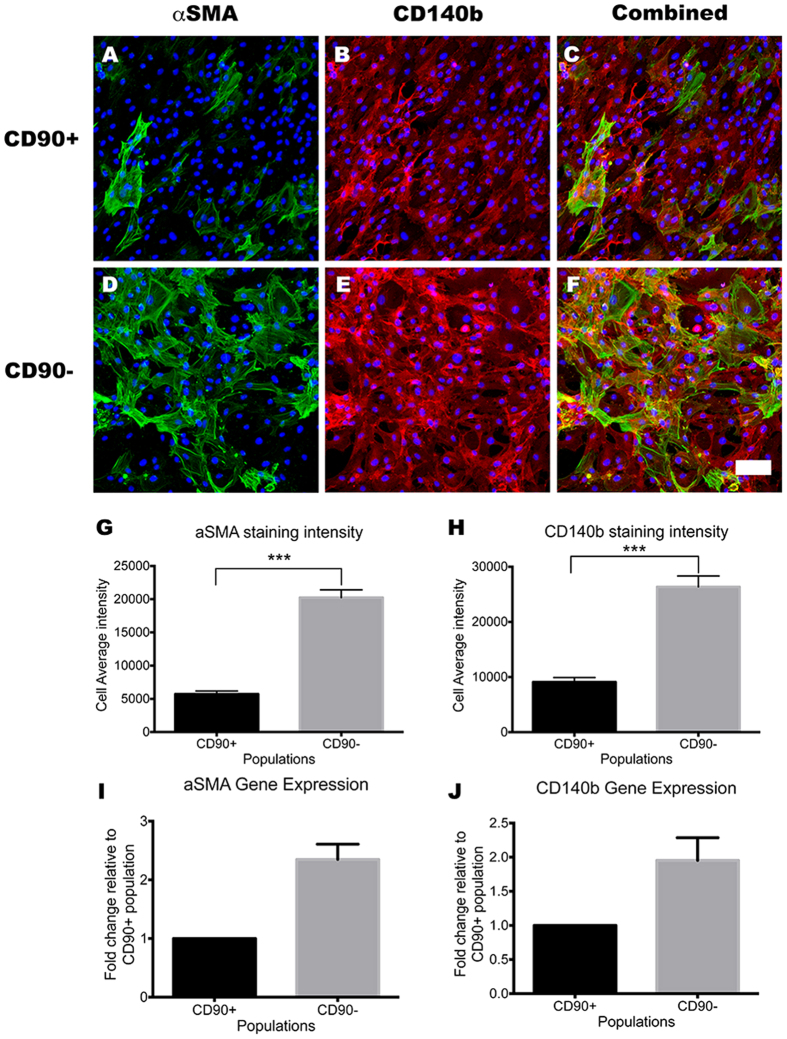
CD90^−^ population expressed higher levels of mature pericyte/myofibroblast markers. Photomicrographs of CD90^+^ and CD90^−^ population immunolabeled for pericyte markers *in vitro* (**A–F**). CD90^−^ population showed greater basal expression of pericyte markers αSMA and CD140b (**D–F**) compared to CD90^+^ population (**A–C**). This was quantified using a cell average staining intensity algorithm and the results are shown in panel G and H (*p* < 0.001). The gene expression analysis using qRT-PCR also supported this, as αSMA and CD140b mRNA levels were at least 2 fold higher in CD90^−^ population relative to CD90^+ ^(**I,J**). Photomicrographs are representative images from 3 separate cases, while image analysis and gene expression data was pooled from at least 4 cases. Scale = 100 μm. ****p <* *0.0001, ***p < 0.001.

**Figure 8:**
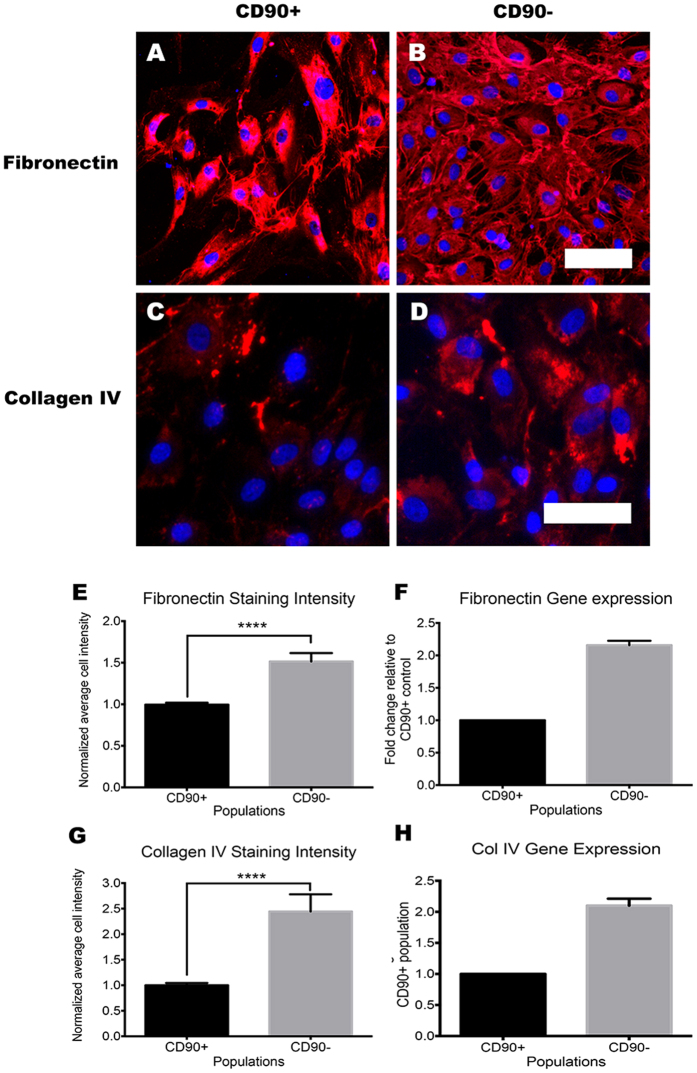
CD90^−^ population expressed greater amounts of extracellular matrix compared to CD90^+^ population. Photomicrographs of CD90^+^ and CD90^−^ population immunolabeled for extracellular matrix components fibronectin and collagen IV (**A–D**). Clearly visible is the larger amount of fibronectin deposited in the CD90^−^ cultures compared to that of the CD90^+^ population (**A,B**) and this was quantified by analyzing the average fibronectin intensity per cell ((**E**) *p* < 0.001). The level of fibronectin mRNA expression corroborated these finding with CD90^−^ having at least twice the basal mRNA expression levels compared to CD90^+^ population (**F**). Collagen IV expression was also higher in the CD90^−^ population, as demonstrated by immunolabeling (**C,D**), image analysis (**G**) and mRNA expression (**H**). Photomicrographs are representative images from three separate cases, while image analysis and gene expression data are pooled from at least three cases. Scale = 100 μm. ****p < 0.0001.

As mRNA levels cannot always predict protein expression, we investigated the protein levels of αSMA, CD140b, collagen IV and fibronectin through immunolabeling and western blot analysis. αSMA and CD140b immunoreactivity were greater in the CD90^−^ cells compared to the CD90^+^ cells (Fig. 7A–F). The 2–3 fold higher expression of the αSMA and CD140b gene in the CD90^−^ population (Fig. 7I–J) resulted in a 3–4 times greater staining intensity per cell when quantified using automated image analysis (Fig. 7G–H; *p* < 0.001). The CD90^−^ population, also expressed greater amounts ECM proteins fibronectin and collagen IV, which are key components of the vascular basement membrane and major proteins produced by neurovascular pericytes. Fibronectin expression was 1.5 times higher, while collagen IV was 2 times higher in the CD90^−^ population compared to that of the CD90^+^ population (Fig. 8). Together, these results not only validate many of our gene expression profiles, but also further consolidate the presence of the higher ECM producing CD90^−^ population.

### The CD90^+^ cells are more sensitive to the actions of TGFβ1

**Figure 9:**
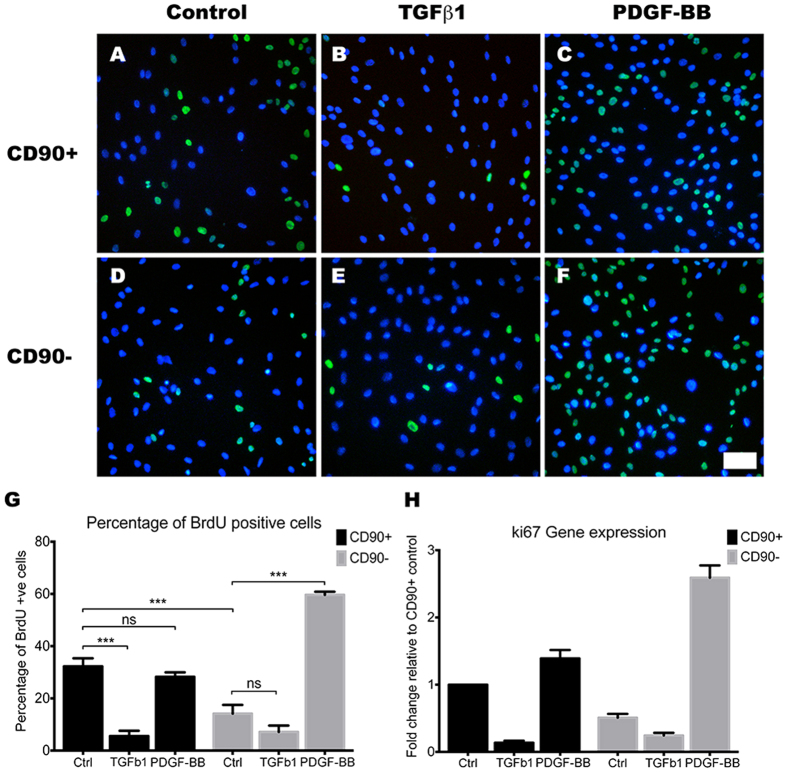
TGFβ1 inhibits proliferation of the CD90^+^ cells, while PDGF-BB stimulates proliferation of the CD90^−^ pericytes. Photomicrographs (**A–F**) of BrdU incorporation (green) overlaid with nuclear Hoechst staining (blue) to show the percentage of BrdU positive cells. BrdU incorporation image analysis (**G**) and ki67 gene expression analysis (**H**) indicates 10 ng/mL TGFβ1 stimulation for 48 hours significantly decreased the proliferation of CD90^+^ cells, while not significantly affecting the CD90^−^ population (*p* < 0.001). In contrast, 48-hour stimulation of 10 ng/mL PDGF-BB greatly increased the proliferation of the CD90^−^ pericyte population while not affecting the CD90^ + ^population (**G,H**; *p* < 0.001). Photomicrographs are a representative case from 3 separate cases while image analysis and gene expression studies are an average of at least 3 different cases. Scale: 100 μm. ***p < 0.001 and ns = *p* > 0.05.

**Figure 10:**
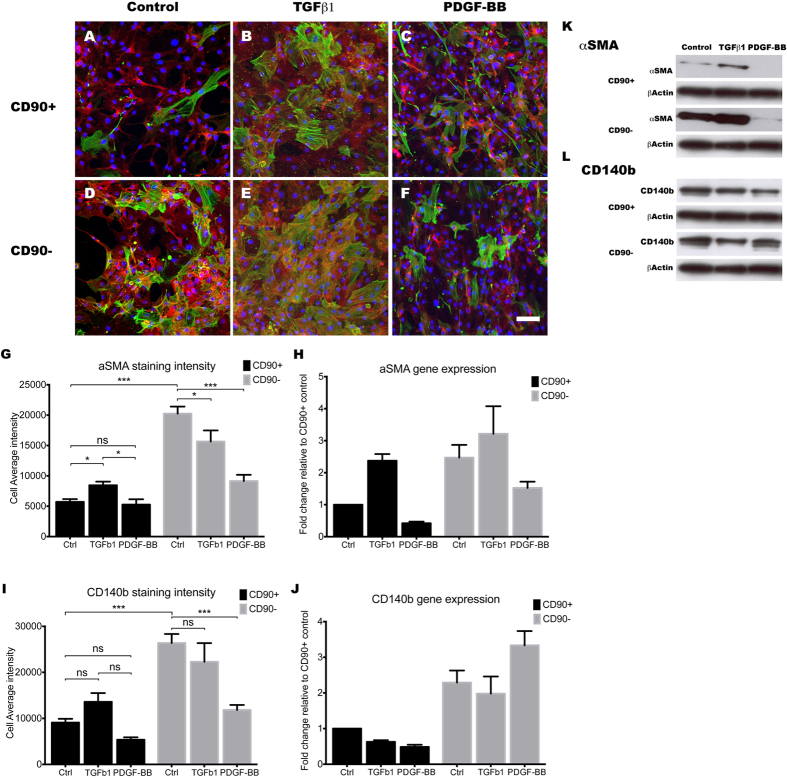
TGFβ1 influences CD90^+^ cells to become more myofibroblast-like, while PDGF-BB induces the CD90^−^ pericytes to express less contractile stress fibres. Photomicrographs (**A–F**) show aSMA (green) overlaid with CD140b (red) along with nuclear stain, Hoechst (blue). As shown earlier, αSMA levels were lower in the CD90^+^ population compared to the CD90^−^ under basal conditions. TGFβ1 clearly increased the expression of αSMA in the CD90^+^ population while not greatly affecting the CD90^−^ population (**A,B,D,E**). The increase in αSMA immunoreactivity in the CD90^+^ population was quantified in panel (**G**) (*p* < 0.05) and reinforced by gene expression (2.5 fold increase (**H**)) and western blot (**K**) analysis. There were no significant changes to CD140b protein or gene expression with TGFβ1 (**A,B,D,E,I,J,L**). In contrast, 10 ng/mL PDGF-BB stimulation for 48 hours resulted in a considerable reduction in the amount of αSMA in the CD90^−^ pericyte population only (**C,F**). This was quantified ((**G**) *p* < 0.001) and reinforced by a 50% reduction in mRNA expression (**H**) and western blot analysis (**K**). Despite the decrease in immunoreactivity for CD140b observed with PDGF-BB stimulation ((**I**) *p* < 0.001), mRNA and western blot analysis both showed no change (**J,L**). In summary, TGFβ1 stimulation seems to affect the CD90^+^ the greatest by increasing myofibroblast/pericyte marker αSMA, while PDGF-BB seems to affect the CD90^−^ population by reverting them back into a more immature phenotype. Photomicrographs are a representative case from 3 separate cases while image analysis and gene expression studies are an average of at least 3 different cases. Scale: 100 μm. ***p < 0.001, **p < 0.01, *p < 0.05 and ns = *p* > 0.05.

TGFβ1 is a potent inducer of pericyte differentiation and adhesion^7^,^49^, and it is implicated in fibrosis and scar formation in the central nervous system^17^,^18^,^50^. For these reasons, we investigated the effects of 48 hours of treatment with 10 ng/mL of TGFβ1 on both CD90^+^ and CD90^−^ population to elucidate their roles in stabilising the neurovascular unit and in scar formation. To our surprise, many differences were observed between the two populations. First, a BrdU assay was used to assess the TGFβ1 effect on cellular proliferation. TGFβ1 stimulation greatly attenuated the proliferation rate of the CD90^+^ population (Fig. 9A,B,G,H; *p* < 0.001), but had very little effect on the CD90^−^ population (Fig. 9D,E,G,H; *p* > 0.05). Furthermore, TGFβ1 stimulation caused a significant increase in αSMA only in the CD90^+^ population (Fig. 10A,B,D,E; *p* < 0.05). However, despite TGFβ1 increasing the levels of αSMA in the CD90^+^ population, the overall levels were still higher in the CD90^−^ population (Fig. 10B,E). CD140b showed no significant changes when induced with TGFβ1 in both populations, showing TGFβ1 does not have a great effect on CD140b expression (Fig. 10I,J). Gene expression levels and immunostaining analysis was further validated by western blot analysis (Fig. 10K,L). Therefore, our data suggests that the more proliferative CD90^+^ cells were more likely to be induced to undergo senescence and differentiate into a mature pericyte or myofibroblast-like cell type, while the more differentiated CD90^−^ population was not further induced by TGFβ1.

### PDGF-BB is a mitogen for CD90^−^ pericytes and also down-regulates myofibroblast-phenotype associated genes

PDGF-BB is another key regulatory cytokine in the formation and maintenance of the BBB^51^. During angiogenesis, PDGF-BB is secreted by endothelial cells to recruit MSCs to the developing vessel and stimulates their differentiation into vascular mural cells^52^,^53^. Furthermore, PDGF-BB is a potent mitogen for the resident pericytes and the vascular smooth muscle cells^52^. Therefore, the response of the two populations to PDGF-BB stimulation was also investigated. Interestingly, unlike the TGFβ1 response, PDGF-BB appeared to stimulate the CD90^−^ population to a greater extent. PDGF-BB stimulation increased BrdU incorporation in the CD90^−^ population, while having no significant effect on the CD90^+^ population (Fig. 9C,F,G). Ki67 gene expression also corroborated this finding with a 5-fold increase in the CD90^−^ population, compared to a 1.2-fold increase in the CD90^+^ population (Fig. 9H). Also, PDGF-BB only affected the CD90^−^ population in terms of αSMA expression, as it decreased αSMA levels to those seen in the CD90^+^ population (Fig. 10C,F). PDGF-BB also decreased the CD140b staining intensity in the CD90^−^ population, although this change was not corroborated by gene expression or western blot analysis (Fig. 10I–L).

### CD90^−^ pericytes show greater inflammatory response to LPS and IFNγ stimulation

**Figure 11:**
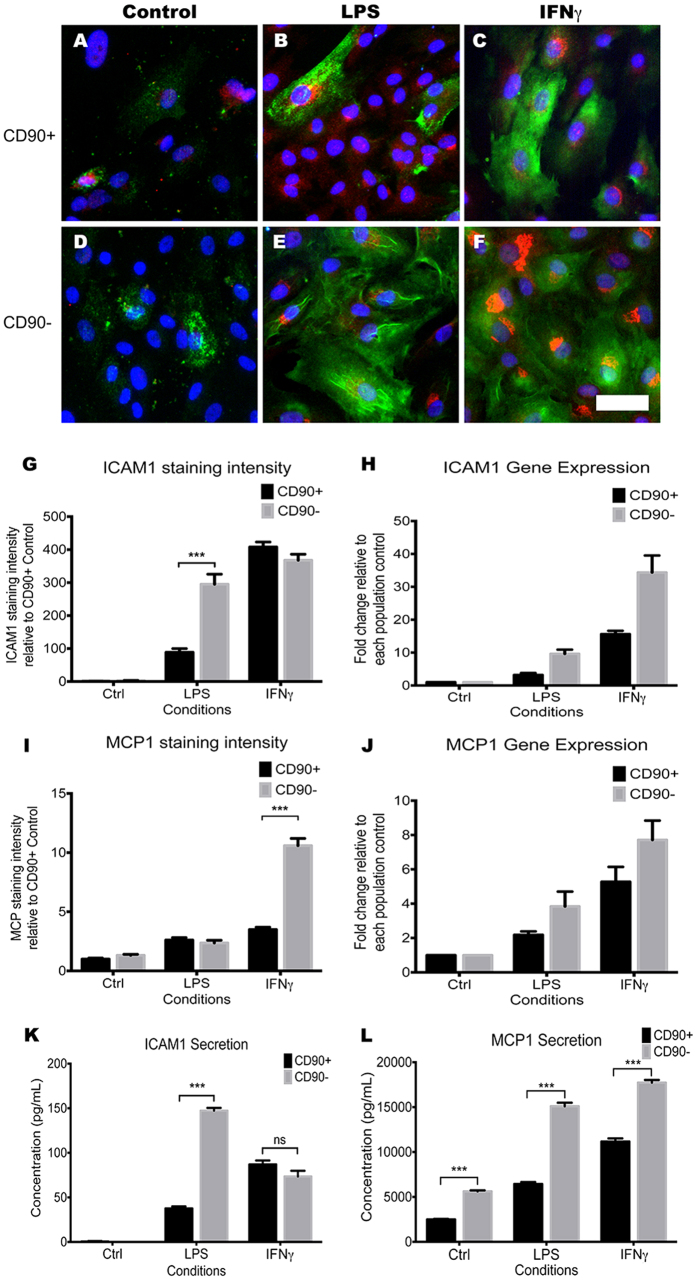
CD90^−^ population shows a greater inflammatory response to LPS and IFNγ. The CD90^−^ population showed significantly higher inflammatory responses when stimulated with 10 ng/mL LPS and IFNγ for 24 hours. Photomicrographs show immunolabeling of pro-inflammatory markers ICAM1 (green) and MCP1 (red) being induced with LPS and IFNγ treatment (**A–F**). Clearly visible is the greater induction of these markers in the CD90^−^ population (**D–F**) in comparison to the CD90^+^ population (**A–C**). Quantification of photomicrographs showed a significant increase in cell staining intensity of ICAM1 with LPS stimulation and MCP1 with IFNγ stimulation (**G,I**). Gene expression data from qRT-PCR also supported these findings (**H,J**). To further this investigation, we studied the secreted inflammatory molecules in the media by using cytometric bead array (CBA). Average of 4 CBA experiments were quantified using a FCAP array and plotted on graphs (**K,L**). The data corroborated our findings showing ICAM secretion being significantly higher in the CD90^−^ population with LPS stimulation (**K**), while MCP1 secretion was higher in every condition in the CD90^−^ populations (**L**). The photomicrographs are representative cases from 3 separate cases while image analysis and CBA analysis were averages of 4 separate experiments. Gene expression analysis is an average of at least 3 separate cases. Scale: 50 μm. ***p < 0.001 and ns = *p* > 0.05.

Previous work from our laboratory^10^ and others^11^,^13^,^54^ have demonstrated that pericytes elicit a strong inflammatory response when stimulated by pro-inflammatory molecules. Therefore, the CD90^+^ and CD90^−^ pericyte populations were investigated for their response to pro-inflammatory molecules. For this, we stimulated both populations for 24 hours with 10 ng/mL lipopolysaccharide (LPS), a bacterial antigen, and a pro-inflammatory cytokine, interferon gamma (IFNγ). We measured their responses by assaying gene expression, protein expression and protein secretion of two well-known pro-inflammatory molecules, intercellular adhesion molecule-1 (ICAM1) and monocyte chemoattractant protein-1 (MCP1). LPS stimulation resulted in an induction of ICAM1 and MCP1 in both populations; however, the response was significantly greater in the CD90^−^ population compared to the CD90^+^ population. Immunocytochemical analysis for ICAM1 and MCP1 was quantified for staining intensity per cell, which clearly demonstrated a greater ICAM1 immunostaining in the LPS treated CD90^−^ population (Fig. 11A,B,D,E,G,I; p < 0.01). By immunostaining, we could not see a difference in MCP1 staining (Fig. 11B,E,I); however, when MCP1 secretion was measured in the culture media using a cytometric bead array (CBA), the CD90^−^ population had a greater level of MCP1 secretion when compared to the CD90^+^ population (Fig. 11L). Soluble ICAM1 secretion, which is also a marker of vascular inflammation^55^, was also higher in the CD90^−^ population (Fig. 11K). This was mirrored by a greater induction of ICAM1 and MCP1 gene expression in the CD90^−^ population in response to LPS treatment (Fig. 11H,J). IFNγ had a similar effect on ICAM1 mRNA expression, increasing it by 24-fold and 15-fold in the CD90^−^ and CD90^+^ populations respectively (Fig. 11H). However, IFNγ-induced cell surface bound and secreted ICAM1 were similar between the two populations (Fig. 11G,K). In contrast, IFNγ-induced MCP1 gene expression, immunostaining, and secretion were similar to those seen with LPS where it was always significantly greater in the CD90^−^ population (Fig. 11C,F,H,J,L,N–P).

The fact that both perivascular cell types up-regulated proinflammatory chemoattractants and cell adhesion molecules in response to two different inflammatory cues, suggest an immunomodulatory role of these cells. However, it is worth noting that the CD90^−^ population consistently showed a greater response. These results support the concept that our brain tissue cultures contain at least two functionally distinct perivascular cell types; one being the more proliferative, naïve CD90^+^ cells, while the other being the more immunologically active, ECM producing CD90^−^ cells.

## Discussion

The main finding of this study is that CD90 can distinguish two functionally related pericyte populations present in the adult human brain. Since pericytes are a key member of the BBB that also regulate cerebral blood flow^8^,^56^, angiogenesis^7^, stabilise the neurovasculature^5^,^6^,^57^, moderate brain inflammatory responses^10^,^11^,^14^, and may be involved in fibrosis and scar formation^18^, these results may have major functional implications on our understanding of the neurovasculature and the BBB.

Although pericytes cultured from temporal lobectomies for intractable complex partial seizures are not from a neurologically intact human brain, our *in vitro* results were highly comparable to those from neurologically ‘normal’ post-mortem brains stained and cultured in our laboratory (un-published findings). Immunohistochemical stains demonstrated the presence of a number of key perivascular markers lining the neurovasculature of our adult human brain specimens. The identification of CD90^+^ cells in the human brain vasculature is intriguing, as prior to this study, it had only been reported in human brain vasculature of glioma specimens^13^,^37^. Previously, another pericyte marker, NG2, was found to be largely located on the arterioles but not on the venules^58^. In conjunction with previous reports that suggest CD90^+^ cells as the immature MSC-like cells in glioma neovasculature^13^,^32^, CD90^+^ cells, like NG2 cells might represent a functionally distinct pericyte population of the neurovasculature.

Furthermore, *in vitro* brain tissue cultures gave rise to two distinct CD90^+^ and CD90^−^ perivascular populations, which when FACS sorted and cultured separately, the CD90^+^ population exhibited higher basal proliferation. In addition, under basal culture conditions, CD90^+^ cells produced lower amounts of ECM proteins and responded less to inflammatory stimuli compared to the CD90^−^ population. In the literature, similar cells have been suggested to be MSCs^11^,^13^,^59^; however, our current study could not definitively state that the CD90^+^ cells are MSCs. Nevertheless, we could clearly distinguish them from the other major population found in our cultures, which were the CD90^−^ population. Extensive phenotypic characterisation of the CD90^−^ population led us to believe that these were the ‘classical’ BBB pericytes reported in the literature^21^, as they exhibited higher levels of classical pericyte markers αSMA and CD140b^20^, and produced higher levels of ECM proteins needed for the formation of the basement membrane.

To examine the possible functional properties of the two populations, we examined the effects of two cytokines, TGFβ1 and PDGF-BB; which are reported to greatly influence the cell fate of the pericytes *in vivo*^7^,^49^,^60^. TGFβ1 is reported to differentiate MSCs and immature pericytes to mature pericytes and/or myofibroblasts when released by the endothelial cells during vessel formation or repair^49^,^60^,^61^ and it is a key cytokine in the wound repair process and scar formation following injury^61^,^62^,^63^,^64^. Therefore, it was interesting to observe that TGFβ1 exerted a relatively small effect on the CD90^−^ population in terms of cellular proliferation contractile protein expression. On the other hand, TGFβ1 had a considerable effect on the CD90^+^ population by: 1) reducing proliferation by over 6-fold to levels found in the CD90^−^ population and 2) increasing their expression of a contractile protein, αSMA. Taken together, it appears that TGFβ1 is influencing the CD90^+^ cells to behave more like the CD90^−^ pericytes.

In addition to vascular stabilisation, TGFβ1 signaling and pericytes have also been implicated in fibrotic scar formation in many organs^2^,^62^,^65^, including the CNS^18^. It is thought that perivascular cells are induced to differentiate into myofibroblasts in response to TGFβ1 activation from injured or inflamed tissue^61^. This not only recruits and differentiates MSCs into myofibroblasts, but also further stimulates the production of ECM^66^. Although our results require further investigation, the fact that TGFβ1 did not affect CD90^−^ pericyte population’s basal ‘fibrotic’ activity, but greatly enhanced CD90^+^ population’s response, suggests a possibility that CD90^+^ perivascular cells are more involved in fibrotic scar formation in the injured brain and spinal cord tissue.

PDGF-BB is another key regulatory cytokine involved in the formation and maintenance of the BBB^51^. Furthermore, PDGF-BB is a potent mitogen for the resident pericytes and the vascular smooth muscle cells^52^. This was evident in our experiments, as PDGF-BB greatly increased proliferation of the CD90^−^ population and dramatically decreased the expression of αSMA (to levels seen in the CD90^+^ population), whilst not affecting any of the above properties in the CD90^+^ population. Whether PDGF-BB acts as a chemo-attractant for these cells will be further elucidated in future studies.

Response to inflammation is a key function of perivascular cells. In conjunction with endothelial cells, pericytes are responsible for the recruitment, adhesion and extravasation of the blood leukocytes^11^,^12^. They also have an immunomodulatory role^10^,^13^, as well as an active phagocytic role^15^. Interestingly, our two pericyte populations showed differing inflammatory response to well-known pro-inflammatory stimuli. When compared to the CD90^+^ population, the CD90^−^ population showed a greater pro-inflammatory response to LPS and IFNγ stimulation in terms of up-regulating ICAM1 and MCP1. ICAM1 up-regulation in response to proinflammatory cues has been well documented in pericytes^10^,^11^,^55^,^67^, and appears to be involved in extravasating peripheral leukocytes into the brain parenchyma^12^. This study also concluded that in mouse skin pericytes, the NG2^+^ pericytes, and not the NG2^−^ pericytes, orchestrated this process^12^. In our study, we could not see a large distinction in NG2 expression between the two populations (Fig. 5); however, the fact that two distinct pericyte-like populations elicited different levels of immunological responses is in line with our data. Furthermore, recent studies provided evidence that the pericytes, but not the MSCs, were mainly responsible for producing an immune response to LPS stimulation, despite appearing phenotypically indistinguishable in the perivascular region^11^,^68^. Moreover, MCP1 expression and secretion were higher basally and in LPS and IFNγ stimulated conditions in the CD90^−^ population (Fig. 11I–L). With a report suggesting that the CD90 molecule inhibits TNFα mediated inflammatory gene expression^69^, it will be interesting to further investigate the lower inflammatory response observed in the CD90^+^ population and whether knocking down CD90 might increase its inflammatory response.

Although there is a possibility of *in vitro* phenotypic switching, our data suggests we have two perivascular populations that are linked in terms of their origin and location but have differing roles in the neurovascular niche. One cell type (CD90^+^) appears to be more naïve in nature and might be primarily responsible for replenishing lost cells, remodeling vasculature during angiogenesis, and possibly involved in scar formation. The other cell type (CD90^−^) appears to be the pericytes that primarily function to maintain the neurovasculature, regulate vessel diameter, and moderate immune responses. Furthermore, a simple phenotypic screen of CD90 was enough to distinguish these two populations *in vitro*, making routine identification and functional studies of these two populations feasible (summarized in Table 1). Currently, studies are underway to further identify the biological significance of CD90 in the neurovasculature. This will allow us to identify possible perivascular population changes in normal and diseased human brains and suggest mechanisms and targets for future therapeutic interventions.

## Methods

### Adult human brain tissue

Human brain tissue containing the anterior temporal lobe and the hippocampus was obtained from surgery for medically refractory epilepsy. All specimens were collected with written patient consent and ethical approval from the Northern X Ethics Committee and the University of Auckland Human Participants Ethics Committee (New Zealand), and in accordance with the approved guidelines. The anterior temporal lobe was used for all the following experiments.

### Immunohistochemistry

Protocols from Waldvogel *et al*.^70^ were used for handling and processing of all donated brain tissue. Briefly, the formalin fixed tissue blocks were coronally sectioned into 50 μm sections and processed for immunohistochemistry as free-floating sections. In cases where antigen-retrieval was necessary, the sections were immersed in 10% formic acid (w/w) for half an hour prior to being incubated for 3 days at 4 °C with the antibodies listed in Table S1. Antibody binding was visualised using DAB or fluorescence and bright field images were imaged using a Leica DMRB microscope (Leica, Germany); confocal images were taken on the Olympus FV1000 microscope (Olympus, Japan).

### Primary perivascular cell culture

Five temporal lobectomy specimens (mean age 26 ± 4 years, n = 5) were collected from the surgical theatre at the Auckland City Hospital. Tissue containing the anterior temporal lobe was mechanically dissected and dissociated prior to being enzymatically digested in HBSS containing 2.5 U/mL papain (Worthington) and 100 U/mL DNase 1 (Invitrogen) for 30 minutes at 37 °C with gentle rotation, which included a gentle trituration step at 15 minutes. Enzymatic digestion was halted by the addition of complete culture media; DMEM:F12 (Invitrogen) containing 10% fetal bovine serum (FBS; Gibco), Penicillin/Streptomycin (Gibco), GlutaMAX (Invitrogen). Cells were collected by centrifugation (170 *g *× 10 minutes), resuspended in the complete culture media and plated onto un-coated T75 culture flasks (Nunc). During the first two days of culture, full media changes were conducted every 24 hours whereby the collected media was centrifuged and cells were resuspended in fresh media and replated into the culture flasks. Thereafter, half media changes were conducted every 2–3 days and cultures were serially passaged upon reaching confluency (typically 20–30 days).

### *In vitro* cytokine treatments

10 ng/mL concentrations were used for all experiments requiring cytokine and pro-inflammatory molecule treatments. Cells were treated for 48 hours with transforming growth factor-beta 1 (TGFβ1) and platelet-derived growth factor-beta (PDGF-BB), while 24-hour treatments were used for lipopolysaccharide (LPS) and interferon gamma (IFNγ).

### FACS and analysis

Upon initial cultures reaching confluency, cells were subjected to FACS and analysis. Adherent cells were detached by a 5 minute incubation with Accutase^®^ (Invitrogen), washed and resuspended in 100 μL of cold staining buffer (PBS with 1% FBS). The cells were stained with a panel of antibodies listed in Table S2 and incubated on ice for 30 minutes. DAPI (1:5,000) was added to determine cell viability. Cells were sorted based on their expression of CD45, CD73 and CD90, and the lack of CD45. Samples were run on a BD SORP FACS Aria II machine (BD Biosciences, CA, USA) and data was analysed using FlowJo VX.0.7 (Treestar). Quadrant markers to determine marker expression were set according to negative control stains, including ‘fluorescence minus one’ (FMO) controls that used all antibodies except that to the marker of interest^25^.

### Immunocytochemistry

Cells were fixed in 4% paraformaldehyde (PFA) for 15 minutes at room temperature and permeabilised by 3 × 10 minute washes in PBS containing 0.1% Triton-X (PBS-T). Antibodies (Table S1) were dissolved in immunobuffer comprising PBS-T with merthiolate and 1% normal goat serum. The cells were stained with primary antibodies listed in Table S1 and incubated overnight at 4 °C, then visualized after a 3-hour room temperature incubation with species-specific fluorescent (Alexa 488 or 594; Invitrogen) or biotin-conjugated (Sigma) secondary antibody. DAB immunoprecipitation was used to visualize biotin-conjugated secondary antibodies and all nuclei were counterstained with 20 μM Hoechst 33258 (Sigma).

### BrdU Assay analysis

10 μM BrdU (Roche) was added to the cultures 24 hours prior to the completion of an experiment. Cells were fixed in ice-cold methanol for 15 minutes at 4 °C followed by a 45-minute incubation in 2 M HCl at 37 °C. The wells were subsequently neutralized by washes in 0.1 M borate buffer pH 8.5 and PBS. Primary BrdU (1:500; Roche) antibody was dissolved in PBS with 1% bovine serum albumin and incubated with cells overnight at 4 °C. Primary antibody visualization was identical to that used for immunocytochemistry.

### Western Blot analysis

Western blots were used to validate results and our primary antibodies. The cell culture medium was removed and the cells were washed twice in ice-cold PBS. Protein lysates were prepared and western blots were performed by protocols previously described^71^.

### Quantitative RT-PCR

Target gene expression levels were evaluated by quantitative RT-PCR using a 7900HT Fast Real Time PCR system (Applied Biosystems, Singapore). Total RNA was isolated at designated time points using the RNeasy kit (Qiagen Inc.) and stored at −80 °C until further use. cDNA synthesis was performed with SuperScript III first strand synthesis kit (Invitrogen) using approximately 3 μg of DNase I-treated (Promega) RNA, and subjected to qRT-PCR using Platinum SYBR Green qPCR SuperMix-UDG with Rox kit (Invitrogen). The primers are detailed in Table S4 and the relative changes were analysed according to the ∆CT method^72^. Each PCR run included a negative RT and non-template control, as well as melting curve assays to confirm specific product amplification. The plotted data represent the mean values of at least 3 independent experiments ± SEM.

### Secreted cytokine measurements using a cytometric bead array

Conditioned media was collected from each of the cell culture conditions at the end of the experiment. To eliminate any debris in the media, the supernatant was collected after centrifugation (500 *g *× 5 minutes) and stored at −20 °C until analysis. The concentrations of cytokines were measured by cytometric bead array (CBA; BD Biosciences, CA, USA; Table S3) as described in^73^. For each cytokine, a 10-point standard curve ranging from 1–5,000 pg/mL was included. The CBA samples were run on an Accuri C6 flow-cytometer (BD Biosciences, CA, USA) and the resulting fluorescent intensities were converted to cytokine concentrations (pg/mL) using the standard curve generated by the FCAP array software (BD Biosciences). This was then calculated into pg per 10^6^ cells from a cell count conducted from the wells in which the media was collected. Details of the flex sets are listed in Table S3.

### Statistical analysis

Unless specified otherwise, all the results were derived from at least 3 independent experiments from at least 3 different brain tissue specimens. Combined or representative data is displayed as mean ± SEM where applicable. Statistical analysis was carried out using student’s t-test for image analysis quantifications comparing CD90^+^ and CD90^−^ populations. Two-way analysis of variance (ANOVA) followed by Bonferroni post-hoc test analysis was used to compare between multiple inflammatory responses. Statistical significance was set at *p* < 0.05. For qRT-PCR data, fold changes of 2 or more were considered significant.

## Additional Information

**How to cite this article**: Park, T. I-H. *et al*. Cultured pericytes from human brain show phenotypic and functional differences associated with differential CD90 expression. *Sci. Rep.* **6**, 26587; doi: 10.1038/srep26587 (2016).

## Supplementary Material

Supplementary Information

## Figures and Tables

**Table 1:** Table summarizing the major phenotypic and functional differences found between the CD90^+^ population and the CD90^−^ population.

Characters/Markers	CD90^+^ Population	CD90^−^ Population
Morphology	Spindle shaped, smaller	Polygonal shape, large and flat
Basal proliferation	High	Low
Phenotypic makers		
CD90	+++	−
αSMA	+	+++
CD140b	++	+++
CD73	+++	+++
CD105	++	+++
CD146	+	+
CD13	+++	+++
Extracellular matrix		
Fibronectin	+	+++
Collagen IV	+	+++
TGFβ1 response		
Proliferation	Decrease	No change
αSMA	Increase	No change
Fibronectin	Increase	No change
PDGF-BB response		
Proliferation	No change	Increase
αSMA	No change	Decrease
Fibronectin	Increase	Increase
Collagen IV	Increase	Increase
Inflammatory response		
LPS-induced ICAM1	+	+++
LPS-induced MCP1	+	+++
IFNγ-induced ICAM1	+++	+++
IFNγ-induced MCP1	++	+++

+++ = High, ++ = Medium, + = Low, − = Very low or Absent.
