# Supplementary material for: Cultured pericytes from human brain show phenotypic and functional differences associated with differential CD90 expression
Source: Sci Rep. 2016 May 24;6:26587. doi: 10.1038/srep26587 (PMC4877602; doi:10.1038/srep26587)
Supplement: Supplementary Information [file srep26587-s1.pdf]

Cultured pericytes from human brain show phenotypic and functional differences associated with differential CD90 expression

Thomas I-H. Park [1,2], Vaughan Feisst [4], Anna E.S. Brooks [4], Justin Rustenhoven [1,2], Hector J. Monzo [1,2], Sheryl X. Feng [1,2], Edward W. Mee [6], Peter S. Bergin [2,6], Robyn Oldfield [5], E. Scott Graham [1,2], Maurice A. Curtis [2,3], Richard L.M. Faull [2,3], P. Rod Dunbar [5], Mike Dragunow [1,2].

Department of Pharmacology and Clinical Pharmacology [1], Centre for Brain Research [2,], Department of Anatomy with Radiology [3], School of Biological Sciences and Maurice Wilkins Centre [4], The University of Auckland, 1023, Auckland, New Zealand; Lab Plus [5], Auckland City Hospital [6], 1023, Auckland, New Zealand.

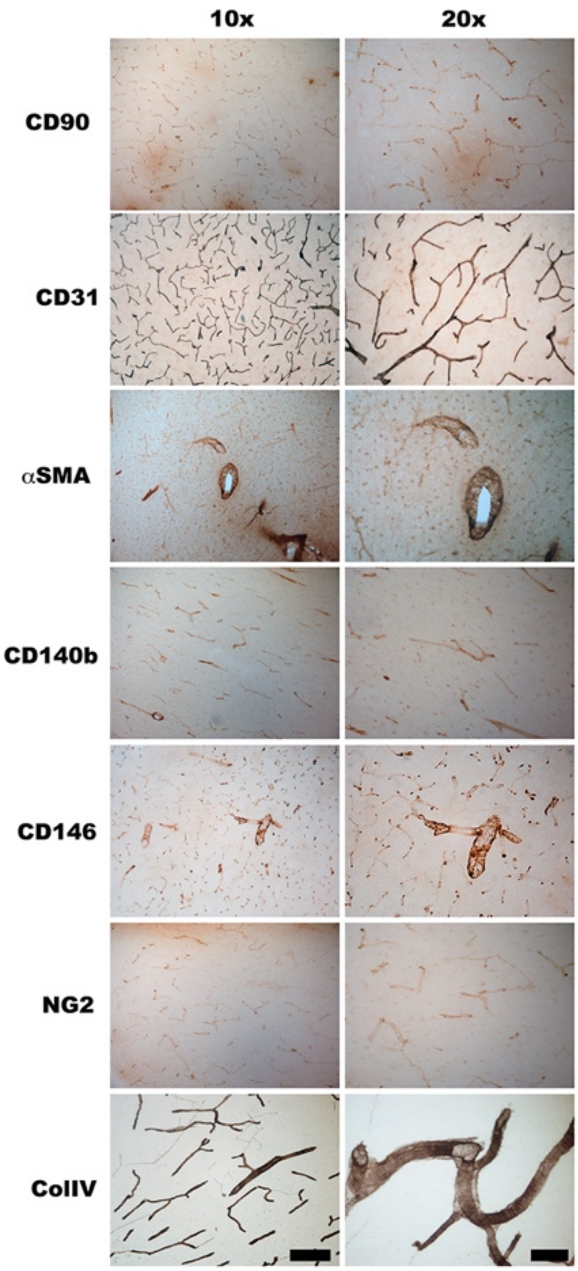

Supplementary Figure 1. Lower magnification images of the key markers used to identify vascular and perivascular cells

Supplementary figure to figure 1 showing photomicrographs of temporal lobe brain sections showing the presence of $CD90^+$ staining in vessels amongst other pericyte markers, αSMA, CD140b, CD146 and NG2. Collagen IV staining shows the basement membrane while CD31 staining shows the endothelial cells of the neurovasculature. Scale: 10x images = 100 µm and 20x images = 50 µm.

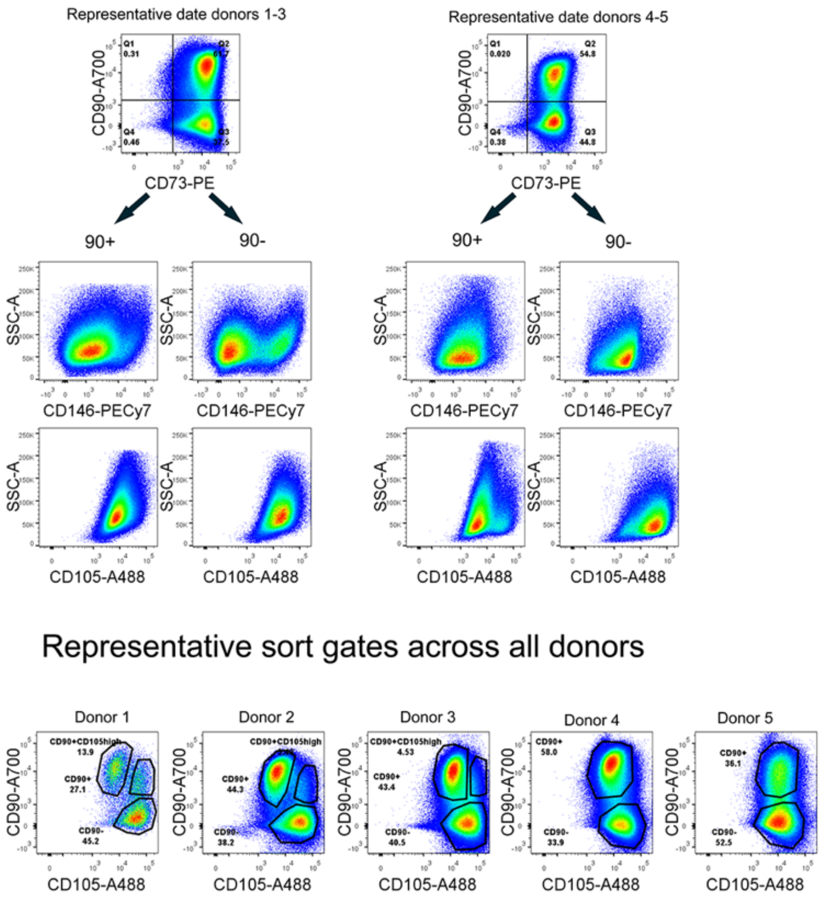

Supplementary Figure 2. Flow cytometry data for 5 cases analysed and sorted for this manuscript

All 5 cases analysed by flow cytometry showed a clear bimodal distribution of the cultured pericytes when gated for CD90. However, there were slight variations in the distribution of CD105 (high and low) in the CD90^{+} population in donors 1 – 3. Furthermore, donors 1 – 3 also showed a slight bimodal distribution of CD146 in the CD90^{-} population. These observations were less evident in donors 4 – 5.

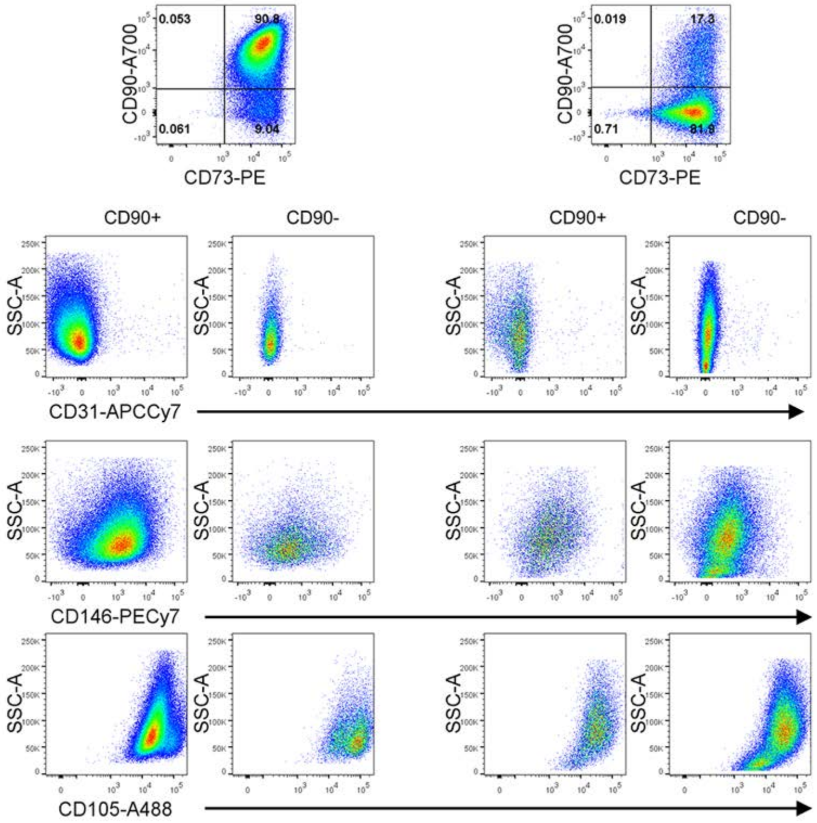

Supplementary Figure 3. Flow cytometry data for a post-sort analysis of CD90 purity after 1 passage in culture

To investigate whether the CD90 phenotype was maintained in culture post-sort, we re-analysed the cell surface phenotype on two of the five cultures after one passage (~10 days). 80 ± 11 % (n=2) of cultured $CD90^+$ population retained CD90 expression, while 82 ± 1.0 % (n=2) of the $CD90^-$ population remained $CD90^-$ for at least one passage. The figure above shows a representative case showing 90.6% of the $CD90^+$ population maintaining CD90 expression while 81.9% of the $CD90^-$ remained $CD90^-$.

Supplementary Table 1. Immunolabeling antibody list

Antibody	Company	Catalog #	ICC dilution	Western dilution	IHC dilutions
αSMA	DAKO	IS611	1:4	1:100	1:4
Beta-actin	Abcam	Ab75186	1:5000	1:10,000	-
BrdU	Roche	13601700	1:500	-	-
Collagen IV	DAKO	M0785	1:500	-	1:500
Fibronectin	DAKO	Q0149	1:1000	1:1000	-
ICAM1	Santa Cruz	sc-107	1:500	1:500	-
ki67	DAKO	M7240	1:500	1:500	-
Prolyl-4-hydroxylase	DAKO	A0245	1:1000	1:1000	-
MCP1	Abcam	Ab9669	1:500	1:500	-
NG2	Santa Cruz	sc-53389	1:1000	1:1000	1:500
CD31	DAKO	M0823	-	-	1:500
CD90	R&D	MAB2067	1:250	1:100	1:100
CD140b	Abcam	Ab32570	1:250	1:500	1:250
CD146	BD	550314	-	1:500	1:500

Supplementary Table 2. Flow cytometry antibody list

Antibody	Company	Catalog #	Dose (μL)/per 10^6 cells (50 μL)
CD13 PerCP Cy5.5	Biolegend	301713	2.5
CD31 APC-Cy7	Biolegend	303120	5.0
CD45 PE CF594	BD	561224	0.6
CD73 PE	BD	550257	5.0
CD90 Alexa 700	Biolegend	328120	1.0
CD105 Alexa 488	Biolegend	323210	5.0
CD140β APC	Biolegend	323608	2.5
CD146 PE-Cy7	Biolegend	342010	5
HLA-DR V500	BD	561225	1.25
Compensation beads	BD	560497	-

Supplementary Table 3. Cytokine bead array antibody list

Antibody	Company	Catalog #	Bead Position
sCD54/ICAM1	BD	560269	A4
MCP1	BD	558287	D8

Supplementary Table 4. List of primers used for qRT-PCR experiments

Gene<br>Acession number	Primer sequence<br>(Forward/reverse)	Start BP	Stop BP	Amplicon Length
b-Actin	TGGTGGGCATGGGTCAGAAGGA	131	152	94 bp
NM_001101	ATGCCGTGCTCGATGGGGTACT	224	203	
Prolyl 4-hydroxylase	TCCAGTTGGGTGATCTGCACCG	653	674	77 bp
NM_001017973	AGCTCGTTCGTGGCTTGGGT	729	710	
ki67	AGCGGAAGCTGGACGCAGAA	7589	7608	79 bp
NM_001145966	TCCAGGGGTTGGGCCTTTTCCT	7667	7646	
Thy-1 (CD90)	AGCAAGGACGAGGGCACCTACA	286	307	68 bp
NM_006288	TGGGAGGAGATGGGTGGGGAAT	353	332	
5'-nucleotidase (CD73)	ATGAACGCCCTGCGCTACGA	313	332	78 bp
NM_002526	TGGCTCGATCAGTCCTTCCACACC	390	367	
PDGFR-B (CD140b)	CGCAAAGAAAGTGGGCGGCT	751	770	80 bp
NM_002609	TGCAGGATGGAGCGGATGTGGT	830	809	
Alanyl Aminopeptidase (CD13)	ACCTGGGTGCTGACTATGCGGA	1256	1277	82 bp
NM_001150	ACTGCCATCACGCGGTACACA	1337	1317	
M-CAM (CD146)	AGTCCCAAGGCAACCTCAGCCA	155	176	76 bp
NM_006500	CGCACACGGAAGATGAGCGT	230	211	
Smooth muscle alpha-actin (a-SMA)	ACGTGGGTGACGAAGCACAGA	164	184	84 bp
NM_001141945	CGTCCCAGTTGGTGATGATGCC	247	226	
NG2	ATGGGAGGTTGGCTTGGCGT	2825	2844	81 bp
NM_001897	GGCCACGCAACAGGTCTTCA	2905	2886	
S100A4	GCCCAGCTTCTTGGGGAAAAGGA	126	148	84 bp
NM_002961	ACCTCGTTGTCCCTGTTGCTGT	209	188	
Collagen Type IV Alpha	CCCGAAAGGCCAGCAAGGTGTT	4134	4155	77 bp
NM_001845.4	GGGCACCGTCAAACCCAGGAAT	4210	4189	
Fibronectin	CGAGAGTGCCCCTACTACAC	4301	4320	84 bp
NM_212482.1	TGTTGGTGAATCGCAGGTCA	4371	4352	
CD105/Endoglin	GGCGGTGGTCAATATCCTGT	1690	1709	111 bp
NM_000118.3	AGGAAGTGTGGGCTGAGGTA	1800	1781	
Nucleostemin (GNL3)	GTGCTGCCAAACTGCTGTGGT	1154	1174	78 bp
NM_014366	GAGTCCAAGATGTAGGGGGATGGC	1231	1208	
